# Sex differences in lipidomic and bile acid plasma profiles in patients with and without coronary artery disease

**DOI:** 10.1186/s12944-024-02184-z

**Published:** 2024-06-26

**Authors:** Benjamin Bay, Marceline M. Fuh, Julia Rohde, Anna Worthmann, Alina Goßling, Natalie Arnold, Lukas Koester, Thiess Lorenz, Christopher Blaum, Paulus Kirchhof, Stefan Blankenberg, Moritz Seiffert, Fabian J. Brunner, Christoph Waldeyer, Joerg Heeren

**Affiliations:** 1 Department of Cardiology, University Heart and Vascular Center Hamburg, University Medical Center Hamburg-Eppendorf, Martinistrasse 52, 20246 Hamburg, Germany; 2 Center for Population Health Innovation (POINT), University Heart and Vascular Center Hamburg, University Medical Center Hamburg-Eppendorf, Hamburg, Germany; 3 German Center for Cardiovascular Research (DZHK), Partner Site Hamburg/Kiel/Luebeck, Hamburg, Germany; 4 Department of Biochemistry and Molecular Cell Biology, University Medical Center Hamburg- Eppendorf, Martinistrasse 52, 20246 Hamburg, Germany; 5 Institute of Cardiovascular Sciences, University of Birmingham, Birmingham, UK; 6 Department of Cardiology and Angiology, BG University Hospital Bergmannsheil, Ruhr- University Bochum, Bochum, Germany

**Keywords:** Atherosclerosis, Cardiovascular disease, Lipidomics, Bile acids, Sex

## Abstract

**Background:**

Lipids, including phospholipids and bile acids, exert various signaling effects and are thought to contribute to the development of coronary artery disease (CAD). Here, we aimed to compare lipidomic and bile acid profiles in the blood of patients with and without CAD stratified by sex.

**Methods:**

From 2015 to 2022, 3,012 patients who underwent coronary angiography were recruited in the INTERCATH cohort. From the overall cohort, subgroups were defined using patient characteristics such as CAD vs. no CAD, 1st vs. 3rd tertile of LDL-c, and female vs. male sex. Hereafter, a matching algorithm based on age, BMI, hypertension status, diabetes mellitus status, smoking status, the Mediterranean diet score, and the intake of statins, triglycerides, HDL-c and hs-CRP in a 1:1 ratio was implemented. Lipidomic analyses of stored blood samples using the Lipidyzer platform (SCIEX) and bile acid analysis using liquid chromatography with tandem mass spectrometry (LC‒MS/MS) were carried out.

**Results:**

A total of 177 matched individuals were analyzed; the median ages were 73.5 years (25th and 75th percentile: 64.1, 78.2) and 71.9 years (65.7, 77.2) for females and males with CAD, respectively, and 67.6 years (58.3, 75.3) and 69.2 years (59.8, 76.8) for females and males without CAD, respectively. Further baseline characteristics, including cardiovascular risk factors, were balanced between the groups. Women with CAD had decreased levels of phosphatidylcholine and diacylglycerol, while no differences in bile acid profiles were detected in comparison to those of female patients without CAD. In contrast, in male patients with CAD, decreased concentrations of the secondary bile acid species glycolithocholic and lithocholic acid, as well as altered levels of specific lipids, were detected compared to those in males without CAD. Notably, male patients with low LDL-c and CAD had significantly greater concentrations of various phospholipid species, particularly plasmalogens, compared to those in high LDL-c subgroup.

**Conclusions:**

We present hypothesis-generating data on sex-specific lipidomic patterns and bile acid profiles in CAD patients. The data suggest that altered lipid and bile acid composition might contribute to CAD development and/or progression, helping to understand the different disease trajectories of CAD in women and men.

**Registration:**

https://clinicaltrials.gov/ct2/show/NCT04936438, Unique identifier: NCT04936438.

**Supplementary Information:**

The online version contains supplementary material available at 10.1186/s12944-024-02184-z.

## Introduction

Atherosclerotic cardiovascular disease (ASCVD) and its manifestation within the coronary wall leading to coronary artery disease (CAD) are the main causes of death in Europe [1]. With regard to the pathophysiological evolution of CAD, low-density lipoprotein cholesterol (LDL-c) and other lipoproteins have been proven to lead to the development of atherosclerosis [2, 3]. The quantification of lipoproteins such as LDL-c and further ApoB-containing lipoproteins can predict ASCVD. In addition, changes in the lipidome, including lipid species such as sterol esters and phospho- and sphingolipids, are associated with the clinical development, presentation and outcome of CAD [4–7]. Quantifying the concentration of various lipids by employing mass spectrometry-based lipidomic analyses provides important insights into the diversity and composition of lipids and lipoproteins [8, 9]. 

Furthermore, rapidly increasing evidence suggests that bile acids (BAs) in combination with atherogenic lipoproteins are potential mediators of ASCVD, including coronary atherosclerosis [10, 11]. More recently, BAs were found to act as hormones mediating the development of ASCVD by signaling through G protein-coupled and nuclear receptors such as FXR, TGR5 and S1PR2, thereby demonstrating their role in health and disease [12]. 

Although some cardiovascular risk factors are similar for both female and male patients, sex-associated differences in the progression of CAD remain poorly understood [13–15]. We therefore aimed to investigate sex differences in the lipidome and BA profiles in a contemporary cohort.

## Methods

### Cohort definition and inclusion and exclusion criteria

The INTERCATH cohort is an observational single-center cohort of patients undergoing coronary angiography at the University Heart and Vascular Center Hamburg-Eppendorf, Germany, and has previously been described in detail [16]. In brief, all patients who underwent coronary angiography at our center were screened for inclusion, and a total of 3,012 individuals were recruited from 2015 to 2021. The study was performed in accordance with the Declaration of Helsinki, and an ethics vote was obtained from the local ethics committee (PV4303, Hamburg, Germany). Moreover, written informed consent was acquired from all participants. The study design and rationale are available at ClinicalTrials.gov (NCT04936438).

For current analyses, a subgroup of patients was selected via the use of a propensity score matching (PSM) algorithm. Details regarding PSM can be found in the statistical analysis section. Patients with myocardial infarction at the time of presentation, previous coronary artery bypass graft surgery, a history of malignancy, intake of any lipid-lowering medication other than statins, cardiac transplantation, or diabetes mellitus type I were excluded. In total, 192 patients were matched. From this cohort, 15 patients were removed from further analysis due to missing blood samples (*n* = 6), prevalent manifest hepatic diseases (*n* = 8), and/or misclassification of CAD (*n* = 1), leaving 177 patients for analysis (the study flow-chart is displayed in Figure S1).

### Assessment of clinical parameters and baseline risk factors

We defined cardiovascular risk factors as follows: age, male sex, diabetes mellitus type II (self-reported/documented), current smoking status (self-reported), body mass index (BMI), and arterial hypertension status (self-reported/documented hypertension or self-reported intake of antihypertensive drugs). The diagnoses were based on patient charts, whereas smoking status was assessed by a standardized questionnaire. The use of statins was either self-reported or documented in patient charts. The established Mediterranean diet score (MDS) was derived as described previously (questionnaire-based assessment of consumption of six food groups and alcohol), aiming to quantify adherence to a Mediterranean diet [17]. Briefly, a score ranging from 0 to 28 points was calculated, where 0 points reflects nonadherence, and 28 points reflects the highest adherence. An MDS of < 12 (failure of MDS) has been proven to be predictive of severity and prognosis for patients with chronic coronary syndromes [21].

### Laboratory measurements and coronary angiography

Blood samples were drawn before coronary angiography. Total cholesterol, triglycerides, high-density lipoprotein cholesterol (HDL-c), LDL-c (measured using the Friedewald formula) and high-sensitivity C-reactive protein (hs-CRP) were determined using standard laboratory measures within clinical practice. Using stored blood samples, high-sensitivity troponin I (hsTnI) concentrations were quantified at our biomarker laboratory utilizing a commercially available immunoassay from Abbott Diagnostics (ARCHITECT STAT).

The obtained coronary angiograms were assessed by interventional cardiologists. In addition to the classical CAD classification, the residual Gensini and SYNTAX scores were calculated as previously published [18, 19].

### Lipidomics

Lipidomic analysis was performed using the Lipidyzer platform™. A triple quadrupole mass spectrometer (QTRAP 5500; SCIEX, Darmstadt, Germany) equipped with a differential mobility spectrometer (DMS) interface operating with SelexION technology was coupled to an ultrahigh-pressure liquid chromatography system (Nexera X2, Shimadzu, Kyoto, Japan) serving as an autosampler. The lipidomics platform (LipidyzerTM) was operated with lipidomics algorithms (Analyst version 1.6.8 and Lipidomics workflow manager; SCIEX). The Lipidyzer™ Platform was tuned using the SelexION Tuning Kit (SCIEX), and a system suitability test was performed using the System Suitability Kit (SCIEX) according to the manufacturer’s instructions. A 50 µL aliquot of plasma from the biobanked blood samples was used for lipid extraction, which was performed as previously described [20–22]. Briefly, plasma samples were spiked with internal standards, and lipids were extracted by employing an adjusted MTBE/methanol extraction protocol [23]. The mass spectrometry-generated data obtained from Analyst software were converted from wiff files to mzML format using MSconvertGUI. Data processing and quantification were performed using the Shotgun Lipidomic Assistant (SLA) software, a python-based application according to Su et al. [23, 24].

### Bile acid measurements

BAs were extracted from the plasma of the biobanked samples by methanol liquid‒liquid extraction. Briefly, 50 µL of plasma was spiked with internal standards, and methanol was added. After vigorous homogenization by vortexing, samples were cleared from debris by centrifugation for 10 min at 10,000 ×g and 4 °C. The supernatants were transferred to fresh tubes and evaporated until dryness. The residual BAs were resuspended in acetonitrile/methanol (3/1, *v/v*) containing 0.1% formic acid and 20 mM ammonium acetate. Quantitative measurement of BAs was performed using an ultrahigh-pressure liquid chromatography system (Nexera X2, Shimadzu, Kyoto, Japan) coupled to a triple quadrupole mass spectrometer (QTRAP 5500; SCIEX, Darmstadt, Germany) (UPLC-QqQ) system operated via Analyst software version 1.6.8 (Sciex). The BAs were separated on a Kinetex C18 column (100 Å, 150 mm × 2.1 mm i.d., Phenomenex, Torrance, CA, USA) run in gradient elution mode with water as eluent A and acetonitrile/methanol (3/1, v/v) as eluent B, both of which contained 0.1% formic acid and 20 mM ammonium acetate. The eluted analytes were ionized in the mass spectrometer with both positive and negative electrospray ionization techniques and analyzed in multiple reaction monitoring (MRM) scan mode. A detailed description of this approach has been previously reported [25]. Peak identification and quantification from the generated chromatograms were performed by comparing retention times, MRM transitions and peak areas to the corresponding standard chromatograms using Multiquant software version 3.0.3 (Sciex). All quantified bile acids are displayed in Supplementary Table S1.

### Statistical analyses

Matching was conducted using the nearest neighbors method, a Mahalanobis distance and a caliper of 0.8, as well as without the reuse of controls and a variable ratio of cases and controls, using R (version 4.1.0) and the “MatchIt” package (version 4.2.0). From the overall cohort, subgroups were defined according to the presence or absence of CAD, 1st vs. 3rd third of LDL-c, and female vs. male sex. Within these subgroups, propensity score matching in a 1:1 fashion was carried out using the following covariables: age, BMI, arterial hypertension status, diabetes mellitus status, current smoking status, MDS status, and the intake of statins, triglycerides, HDL-c, and hs-CRP. For each sex, further analyses were conducted with the first LDL-c third as the reference, stratified by the presence or absence of CAD.

Categorical variables are shown as absolute numbers and percentages and were compared by Fisher’s exact test. Continuous variables are described as medians, 25th percentiles and 75th percentiles and were compared by the Mann‒Whitney test. Baseline plasma lipid and BA characteristics were compared using the Kruskal‒Wallis test. For visualization of lipid concentration/composition in the subgroups using volcano plots, the negative logarithm of the *p* value calculated by Student’s t test on the base 10 is plotted against the logarithm of the fold change between the two conditions. The concentration and composition of all lipid species investigated, the fold changes and *p* values are given in the Supplementary Tables. A two-sided *P* value of < 0.05 was considered to indicate statistical significance. All statistical analyses, including volcano plot analysis, were carried out utilizing R statistical software, version 4.1.0 (R Foundation for Statistical Computing), GraphPad Prism, version 10.3.2, and Excel 2016.

## Results

### Baseline characteristics

After propensity score matching, 177 patients were included in the current analysis. The median ages were 73.5 years (25th and 75th percentiles: 64.1, 78.2) and 71.9 years (65.7, 77.2) for females and males with CAD, respectively, and 67.6 years (58.3, 75.3) and 69.2 years (59.8, 76.8) for females and males without CAD, respectively. Across the subgroups, all baseline characteristics, including classical cardiovascular risk factors such as diabetes, intake of statins, and crude lipoprotein levels such as LDL-c, were evenly distributed. Detailed information about baseline characteristics is provided in Table 1 and Supplementary Table S2 (stratified according to sex, presence or absence of CAD and LDL-c third).

**Table 1:** Baseline characteristics

	CAD		No CAD		
	Women (*n* = 44)	Men (*n* = 44)	Women (*n* = 44)	Men (*n* = 45)	*p* Value
**Demographics and comorbidities**					
Age (years)	73.5 (64.1, 78.2)	71.9 (65.7, 77.2)	67.6 (58.3, 75.3)	69.2 (59.8, 76.8)	0.17
BMI (kg/m2)	26.6 (23.5, 30.8)	26.3 (24.1, 28.3)	24.6 (21.9, 28.2)	26.3 (24.4, 30.4)	0.31
Hypertension (%)	42 (95.5)	42 (95.5)	42 (95.5)	40 (88.9)	0.56
Diabetes mellitus (%)	4 (9.1)	4 (9.1)	2 (4.5)	3 (6.7)	0.83
Current smoking (%)	8 (18.2)	4 (9.1)	5 (11.4)	4 (8.9)	0.52
MDS (points)	13.0 (11.3, 14.7)	13.7 (12.0, 14.5)	13.0 (10.8, 14.1)	13.0 (11.3, 14.7)	0.67
Intake of Statins (%)	12 (27.3)	9 (20.5)	10 (22.7)	7 (15.6)	0.60
**Laboratory values**					
Total Chol. (mg/dL)	162.0 (136.8, 197.5)	169.0 (138.0, 201.2)	181.0 (140.2, 203.0)	164.0 (117.0, 196.0)	0.35
HDL-c (mg/dL)	54.5 (46.0, 62.2)	52.5 (42.8, 59.0)	54.5 (45.0, 62.2)	48.0 (41.0, 64.0)	0.60
Triglycerides (mg/dL)	105.5 (78.0, 116.2)	83.0 (73.0, 127.5)	90.5 (70.5, 135.0)	86.0 (65.0, 110.0)	0.28
LDL-c (mg/dL)	75.0 (63.5, 121.2)	112.0 (58.8, 122.2)	92.0 (62.0, 126.5)	74.0 (59.0, 124.0)	0.84
hs-CRP (mg/L)	0.2 (0.1, 0.7)	0.3 (0.1, 0.9)	0.4 (0.1, 0.8)	0.2 (0.1, 0.6)	0.72
hsTnI (ng/L)	6.4 (2.3, 13.2)	7.3 (2.7, 17.8)	2.9 (1.4, 11.0)	4.6 (2.3, 8.3)	0.24
**CAD classification**					
Gensini Score	8.0 (2.5, 19.6)	14.0 (5.0, 42.0)	-	-	-
Syntax Score	6.5 (0, 10.2)	8.0 (0, 20.0)	-	-	-

Categorical variables are shown as absolute numbers and percentages and were compared by Fisher’s exact test. Continuous variables are described as medians and 25th percentiles/75th percentiles and were compared by the Mann‒Whitney test. *BMI* body mass index; *CAD* coronary artery disease; *HDL-c* high-density lipoprotein cholesterol; *hs-CRP* high-sensitivity C-reactive protein; *hsTnI* high-sensitivity troponin I; *MDS* Mediterranean diet score; *LDL-c* low-density lipoprotein cholesterol (measured using the Friedewald Formula); *Total Chol*. Total cholesterol

### Lipid class characteristics

**Fig. 1:**
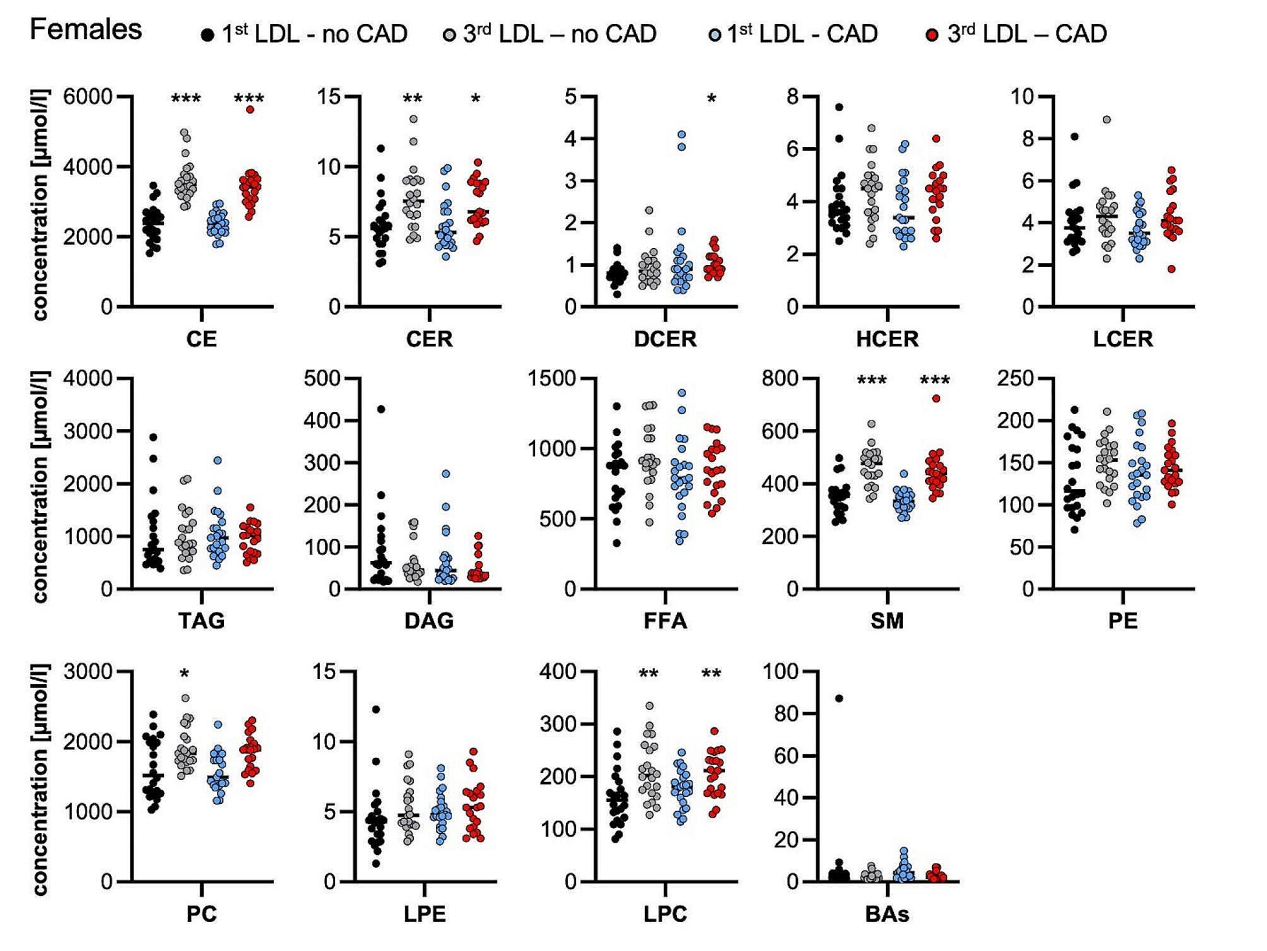
Baseline lipid class characteristics in females. Lipid classes were quantified in plasma by mass spectrometry. Individual data points are presented in µMol/l, and vertical lines represent median values. CE: cholesteryl esters, CER: ceramides, DAG: diacylglycerols, FFA: free fatty acids, HCER: hexosylceramides, LCER: lactosylceramides, LPC: lysophosphatidylcholines, LPE: lysophosphatidylethanolamines, PC: phosphatidylcholines, SM: sphingomyelins, TAG: triacylglycerols, BA: bile acids. *P < 0.05, ** *P* < 0.01, *** *P* < 0.001 compared to the 1st third LDL-no CAD group by the Kruskal‒Wallis test

**Fig. 2:**
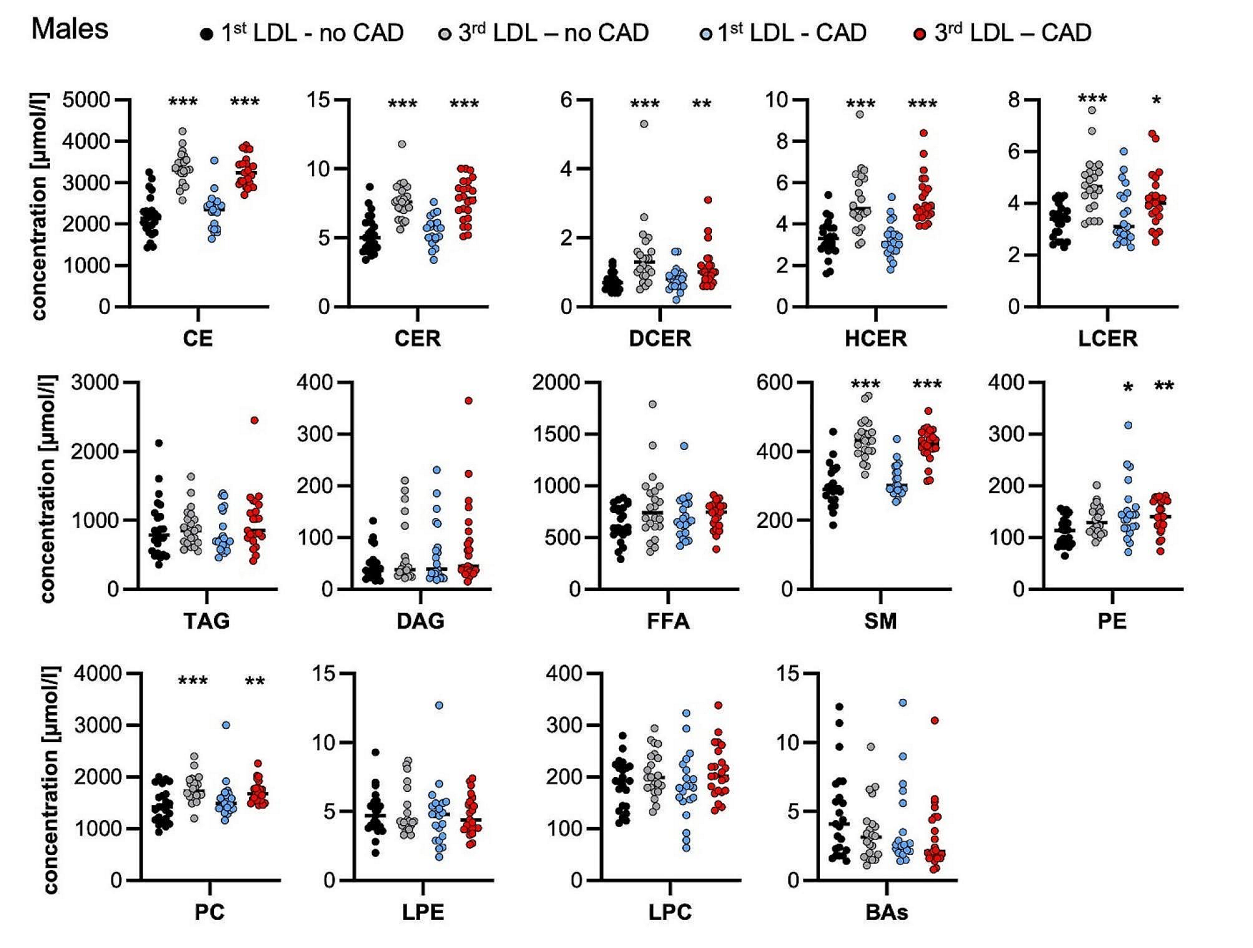
Baseline lipid class characteristics in males. Lipid classes were quantified in plasma by mass spectrometry. Individual data points are presented in µMol/l, and vertical lines represent median values. CE: cholesteryl esters, CER: ceramides, DAG: diacylglycerols, FFA: free fatty acids, HCER: hexosylceramides, LCER: lactosylceramides, LPC: lysophosphatidylcholines, LPE: lysophosphatidylethanolamines, PC: phosphatidylcholines, SM: sphingomyelins, TAG: triacylglycerols, BA: bile acids. * *P* < 0.05, ** *P* < 0.01, *** *P* < 0.001 compared to the 1st third LDL-no CAD group by the Kruskal‒Wallis test

Analysis of 13 lipid classes at baseline (CE: cholesteryl esters, CER: ceramides, DAG: diacylglycerols, FFA: free fatty acids, HCER: hexosylceramides, LCER: lactosylceramides, LPC: lysophosphatidylcholines, LPE: lysophosphatidylethanolamines, PC: phosphatidylcholines, SM: sphingomyelins, TAG: triacylglycerols) revealed a similar pattern in lipid class concentrations in the female and male subgroups. In line with the selection criteria that were based on LDL-c plasma levels, for both female and male patients within the 3rd LDL-c third, a significantly higher concentration of cholesteryl esters was observed. No significant differences in BA concentrations were noted between the sexes. The detailed baseline lipid class characteristics are included in Fig. 1 (females) and Fig. 2 (males).

**Fig. 3:**
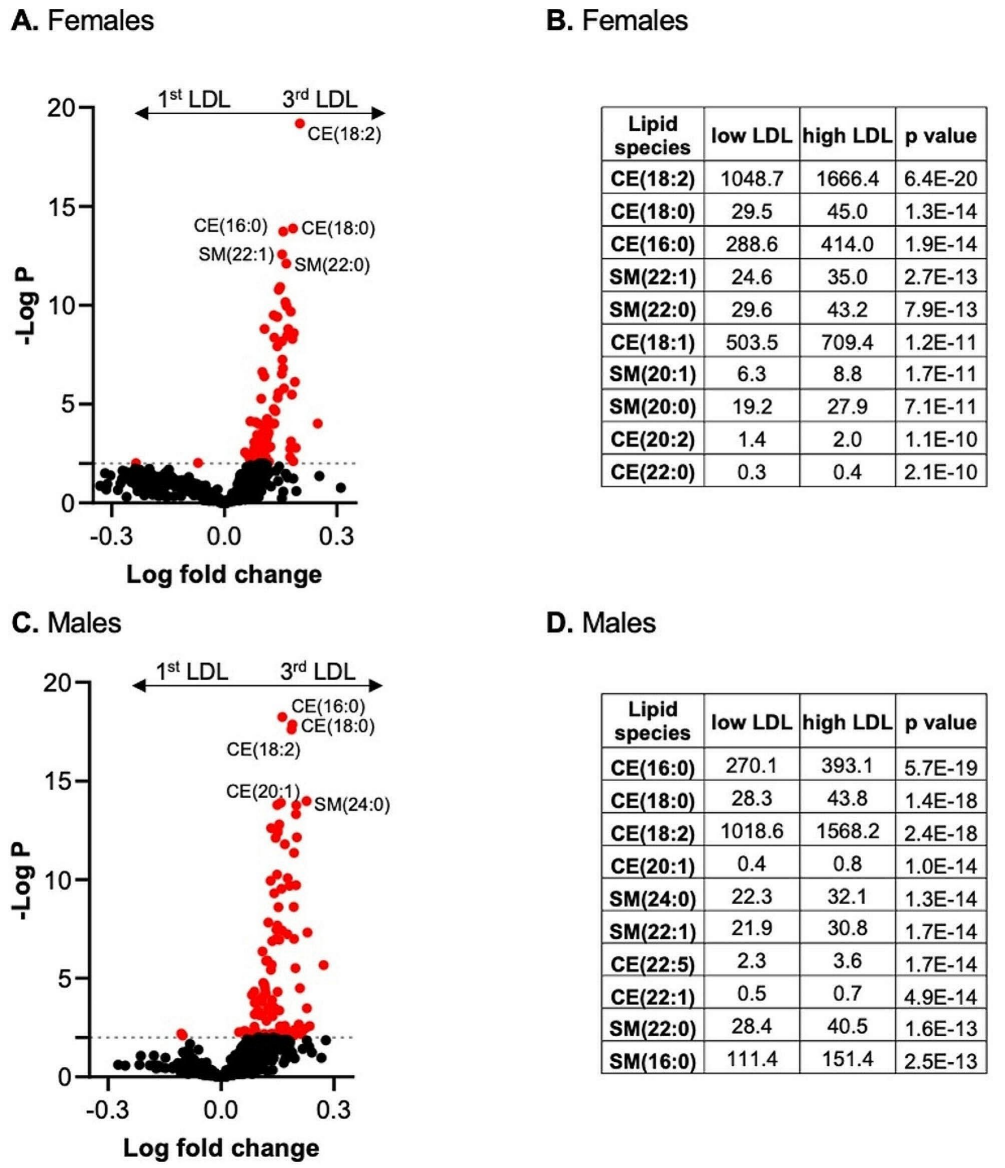
Volcano plot for plasma lipid and bile acid species in comparison between patients with low and high LDL-c. (**A**, **C**) The x-axis represents the log fold change, and the y-axis represents the –log10 *p* value. Values of differentially abundant lipid species with *p* < 0.01 (-log *P* > 2) in (**A**) females and (**C**) males are highlighted in red. (**B**, **D**) Plasma levels of the ten most significantly different plasma lipid species in the (**B**) female subgroup and (**D**) male subgroup

A volcano plot of differentially altered lipid species revealed significantly more cholesterol ester species, such as CEs (18:2), when comparing women (Fig. 3A-B) to men (Fig. 3C-D) in the 1st versus 3rd LDL-c tertiles. In addition to the cholesteryl ester species, a number of sphingomyelin species were substantially more abundant in both females and males in the third LDL-c group. Overall, the grouping of patients according to LDL-c levels was confirmed by lipidome analysis, which demonstrated the validity and reliability of the methodology used. All analyzed lipid and BA species and the respective concentrations as well as *p* values used for Fig. 3 are included in the Supplemental Table for Fig. 3.

### CAD-associated differences in plasma lipid and bile acid concentrations and composition in women

**Fig. 4:**
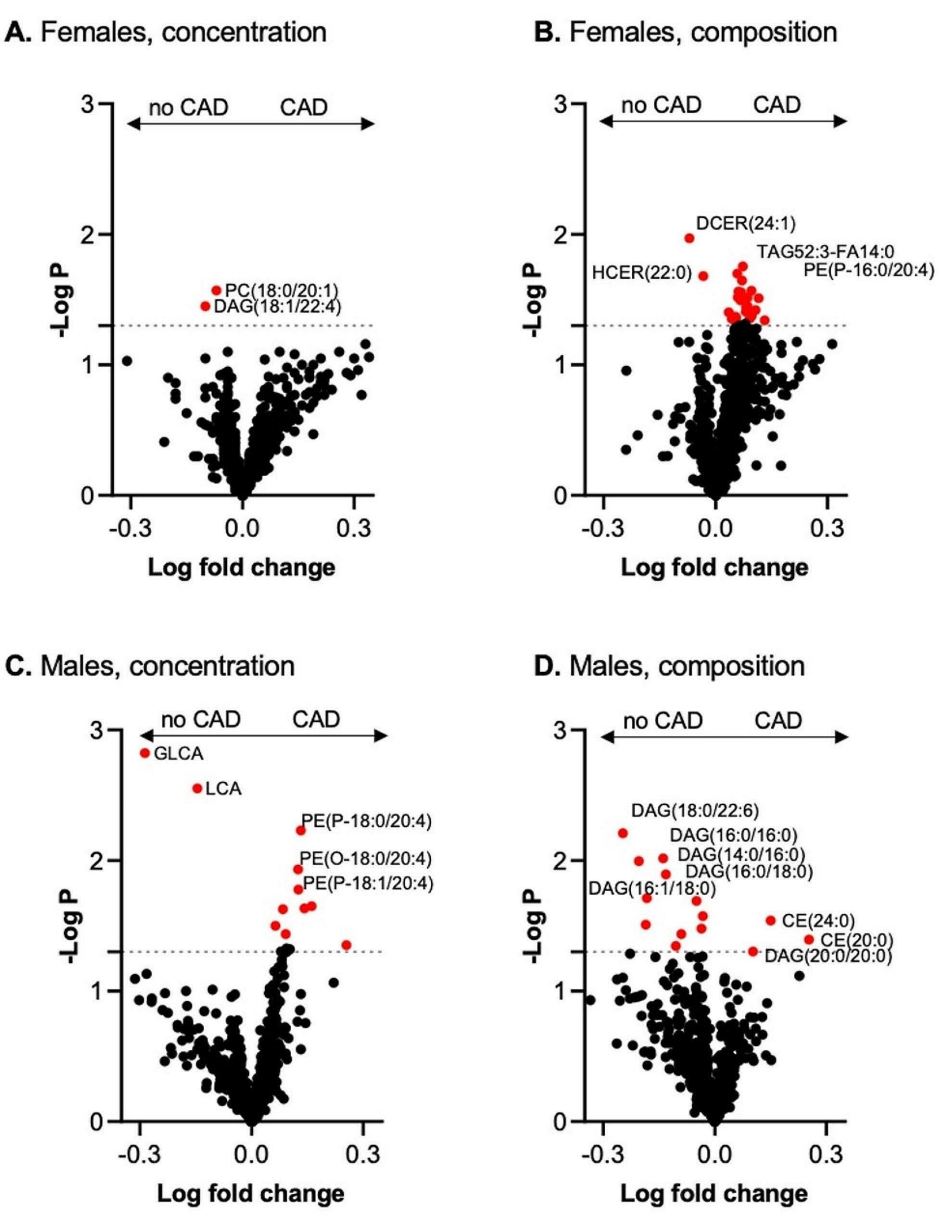
Volcano plot for plasma lipid and bile acid species concentrations and compositions in patients with and without CAD. The x-axis represents the log fold change, and the y-axis represents the –log10 *p* value. (**A**) Differentially abundant lipid species in females with an FDR > 1.3 are highlighted in red. (**B**) Differentially abundant lipid species in females with an FDR > 1.3 are highlighted in red. (**C**) Differentially abundant lipid species in males; FDRs > 1.3 are highlighted in red. (**D**) Differentially abundant lipid species in females; FDRs > 1.3 are highlighted in red

**Fig. 5:**
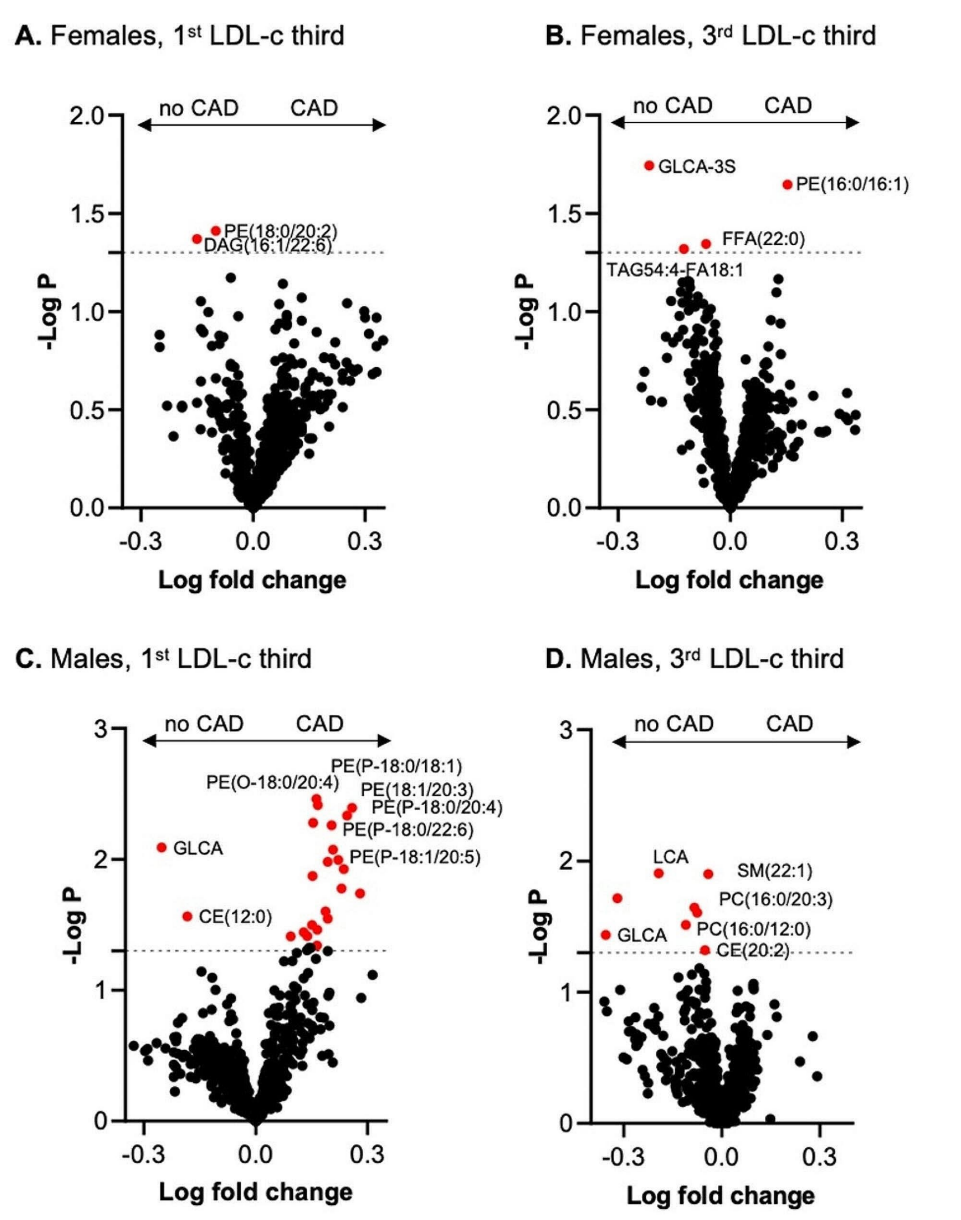
Volcano plot for plasma lipid and bile acid species concentrations according to LDL-c thirds in patient subgroups without and with CAD. The x-axis represents the log fold change, and the y-axis represents the –log10 *p* value. Values of *p* < 0.05 (-log *P* > 1.3) are highlighted in red. Differentially abundant lipid species (**A**) in females in the 1st LDL-c third group, (**B**) in females in the 3rd LDL-c third group, (**C**) in males in the 1st LDL-c third group, and (**D)** in males in the 3rd LDL-c third group

PC(18:0/20:1) and DAG(18:1/22:4) were lower in women with CAD than in women without CAD (Fig. 4A). Furthermore, the lipid components HCER (22:0) and DCER (24:1) were lower, and TAG52:3-FA14:0 and PE (P-16:0/20:4) were greater in women with CAD than in those without CAD (Fig. 4B), while no differences in the BA profile were noted according to the presence or absence of CAD. Further analysis suggested that plasma lipid levels did not differ among women in the subgroup with high LDL-c levels (Fig. 5A-B). All analyzed lipid species and BA species and the respective concentrations as well as *p* values used for Figs. 4 and 5 are included in the Supplemental Table for Figs. 4 and 5, respectively.

### CAD-associated differences in plasma lipid and bile acid concentration and composition in men

The lipid components DAG(18:0/22:6), DAG(16:0/16:0), DAG(14:0/16:0), DAG(16:0/18:0) and DAG(16:1/18:0) were lower, and CE(24:0), CE(20:0) and DAG(20:0/20:0) were greater in men with CAD than in men without CAD (Fig. 4C-D). In contrast to those in women, the concentrations of the secondary BAs glycolithocholic acid (GLCA) and lithocholic acid (LCA) were lower, and the concentrations of PE (P-18:0/20:4), PE (O-18:0/20:4) and PE (P-18:1/20:4) were greater in men with CAD than in those without CAD (Fig. 4C-D). Several plasmalogen lipids (PE-P) were greater in men with low LDL-c (Fig. 5C), an effect not observed in males with high LDL-c (Fig. 5D).

## Discussion

In our study, a comparison between female and male CAD and non-CAD patients accounting for possible confounders through the implementation of a matching algorithm revealed a sex-specific pattern of both lipidomic and BA profiles. Although females with CAD had no changes in BA concentrations in contrast to non-CAD females, males with CAD had significantly lower levels of the secondary BAs GLCA and LCA than the control group without CAD. These hypothesis-generating findings identify potential drivers of sex-specific differences in the development of CAD.

In recent years, population-based studies have shown sex differences in both metabolic and lipidomic parameters [26, 27]. Recently, in a study from Tabassum and colleagues, sex-specific changes in 141 lipid species were demonstrated. This finding was shown to be age dependent since opposite age-related changes were observed in 39 lipid species, and sex differences were attenuated with increasing age. Interestingly, and confirmatory of our results concerning the reported changes in some lipid levels, Tabassum et al. demonstrated that both age- and sex-associated changes in lipid concentrations and composition extended to PCs, PEs and CER, which were deflected in our study cohort [28]. 

Concerning lipids and lipoproteins, we reported downregulated concentrations of PC (18:0/20:1) in female patients with CAD, while no changes in PCs were noted in male patients. The predictive utility of circulating PCs has been demonstrated via integration in the so-called ceramide test score (CERT2), which consists of one ceramide/ceramide ratio, two ceramide/PC ratios and a single PC. In contrast to our results, during the development of the CERT2 score, PC (14:0/22:6), PC (16:0/22:5) and PC (16:0/16:0) were identified to predict cardiovascular outcomes [29]. Various associations of PC-esters have been described in the literature, with both elevated and decreased levels of PC being associated with CAD [6, 30]. Hence, the broader utility of PCs in cardiovascular disease manifestation still needs to be clarified further. Interestingly, ceramides, including DCER (24:1), were significantly decreased in terms of lipid composition in female CAD patients in our study. This is in contrast to a previous report in which the levels of all sphingolipids measured, except for 3 glucosylceramides, were elevated in patients with CAD, although no sex-specific analysis was performed [5]. Similarly, between the sexes, DAG levels were lower in both subgroups without CAD. DAGs have been described as components of cellular membranes, as well as lipid second messengers with signaling properties linked to metabolism [41]. However, previous reports have noted inconsistent results with regard to the impact of DAG, as both up- and downregulated levels of DAG have been described in different disease entities [4, 7, 31, 32]. 

In this study, we found higher plasmalogen levels specifically in male CAD patients with low LDL-c levels. Chronic inflammatory diseases have been reported to correlate with lower plasmalogen levels, which is contrary to our observation [33]. On the other hand, plasmalogens can initiate both anti- and proinflammatory responses. The latter seems to be associated with the release of arachidonic acid, which, in turn, serves as a precursor to produce proinflammatory lipid mediators [33]. Additional studies will be necessary to mechanistically decipher the relevance of plasmalogens for CAD progression, especially in the context of hypo- and hypercholesterolemic conditions.

In recent years, a multitude of potential pathways/mechanisms related to sex-specific changes in the lipidome have been investigated. These include the effects of sex hormones (estrogen and testosterone through various signaling pathways), autosomal genetic variants, incomplete X chromosome inactivation, and male-specific Y chromosome mutations, as well as external factors, including diet and physical activity [15]. However, the precise mechanisms underlying sex-specific lipid profiles remain to be elucidated.

Moreover, in our study, no BA perturbations according to CAD status were detected in female patients. However, specific changes in the secondary BAs GLCA and LCA were noted in the male CAD subgroup. Recently, BAs have been investigated as potential mediators in the development of atherosclerosis, with lower serum BA concentrations being present in patients with CAD in both large-scale cohorts and smaller studies [11, 34]. Here, lower total BA concentrations were associated with CAD, MI and the severity of coronary lesions, as measured by the Gensini score, irrespective of patient sex [34]. More recently, Nguyen and colleagues confirmed the association of lower BA serum concentrations in patients with CAD, and it was demonstrated that glycochenodeoxycholic acid, a conjugated primary BA, was predictive of CAD [11]. 

One of the potential atheroprotective effects of BAs has been hypothesized to be attributed to their signaling properties through molecular action targeting the TGR5 and FXR receptors, potentially via anti-inflammatory or direct metabolic effects [35, 36]. Building on these observations, BA receptors have been used as therapeutic target in patients with steatohepatitis, where obeticholic acid, a receptor ligand for the farnesoid X nuclear receptor, has been shown to improve histological measures [37, 38]. Hence, our finding that changes in BA patterns are sex specific has potential clinical implications for these newly developed methods in broader applications, including in cardiovascular disease [39]. 

Our study did not provide mechanistic insights into the observed differences in BA metabolism. However, differences in the BA pool and synthesis have been described in other disease entities [40, 41]. Here, the effects of synthetic and endogenous estrogens have been implicated in the observed group differences both for cardiovascular and other disease manifestations [14, 42, 43]. Additionally, as LCA and GLCA are secondary BAs and are thus derived from gut microbiota, the changes in their concentrations might also be products of altered gut flora in CVD patients.

Although prior studies have investigated either the lipidome or BA in patients with CAD, we believe that several aspects of our work are novel. First, we carried out parallel quantification of the lipidome as well as BAs in our cohort, aiming to unravel the potential interplay between these mediators of atherosclerosis. Second, using a propensity score matching algorithm, we aimed to account for a multitude of confounding factors for both lipid and BA levels, which are commonly not considered in other studies [44, 45]. Finally, our results represent the first sex-specific analysis of the lipidome and BAs in patients with CAD in comparison to a non-CAD control cohort.

### Study strengths and limitations

Strengths of the study include angiographic assessment for coronary artery disease in all patients and state-of-the-art quantification of lipid and bile components in their blood. Although we describe a very well-defined contemporary all-comers CAD cohort with broad matching criteria, some limitations merit consideration. First, the number of patients was low, limiting our ability to correct for multiple testing. Second, serial measurements of BAs at different timepoints and lipidomic profiles were not carried out. Natural variations in the concentrations of the measured components were therefore not captured. Additionally, while our patient cohort was precisely characterized concerning CAD severity and baseline characteristics, selective screening with regard to potential confounding disease entities such as steatohepatitis and fatty liver disease, which have been shown to be associated with circulating BA levels, was not implemented. However, using a matching algorithm, we aimed to account for metabolic abnormalities, including diabetes and obesity, which are associated with the development of liver disease, limiting the confounding potential of these disorders. Finally, due to the relatively small sample size in our present investigation, which accounts for a limited proportion of the overall INTERCATH cohort, our current analysis delivers hypothesis-generating data only. Additionally, the identification of lipidomic or bile acid predictors of CAD is limited and requires further exploration in larger-scale studies.

## Conclusion

Our study adds to the growing body of literature showing that a sex-specific pattern in circulating lipid species as well as BAs is present in patients with ASCVD. To further understand the potential causative changes in the sex-specific pathophysiology of atherosclerosis, mechanistic studies are needed. Nonetheless, our findings have clinical implications concerning the utilization of lipidomic measurements in the risk prediction of patients with cardiovascular disease and the potential targeting of BA receptors as treatment in patients with CAD.

### Electronic supplementary material

Below is the link to the electronic supplementary material.

Supplementary Material 1

## Data Availability

The dataset supporting the conclusions of this article is included within the article and its additional files.

## Abbreviations

ASCVD: Atherosclerotic cardiovascular disease
BA: Bile acid
BMI: Body mass index
CAD: Coronary artery disease
CE: Cholesteryl esters
CER: Ceramides
DAG: Diacylglycerols
FFA: Free fatty acids
GLCA: Glycolithocholic acid
HCER: Hexosylceramide
HDL-c: High-density lipoprotein cholesterol
hs-CRP: High-sensitivity C-reactive protein
hsTnI: High-sensitivity troponin I
LCER: Lactosylceramide
LDL-c: Low-density lipoprotein cholesterol
LPC: Lysophosphatidylcholine
LPE: Lysophosphatidyethanolamines
MDS: Mediterranean diet score
PC: Phosphatidylcholine
PSM: Propensity score matching
SM: Sphingomyelins
TAG: Triacylglycerols
LCA: Lithocholic acid

## Declarations

### Previous presentation

Part of this work has previously been presented at the 91st Congress of the European Atherosclerosis Society, 21st -23rd May 2023, Mannheim, Germany.
