# Supplementary material for: Sex differences in lipidomic and bile acid plasma profiles in patients with and without coronary artery disease
Source: Lipids Health Dis. 2024 Jun 26;23:197. doi: 10.1186/s12944-024-02184-z (PMC11201360; doi:10.1186/s12944-024-02184-z)
Supplement: Supplementary file 1 — Supplementary Material 1 [file 12944_2024_2184_MOESM1_ESM.docx]

**Supplementary appendix**

***Figure S1: Study Flowchart****.*

**
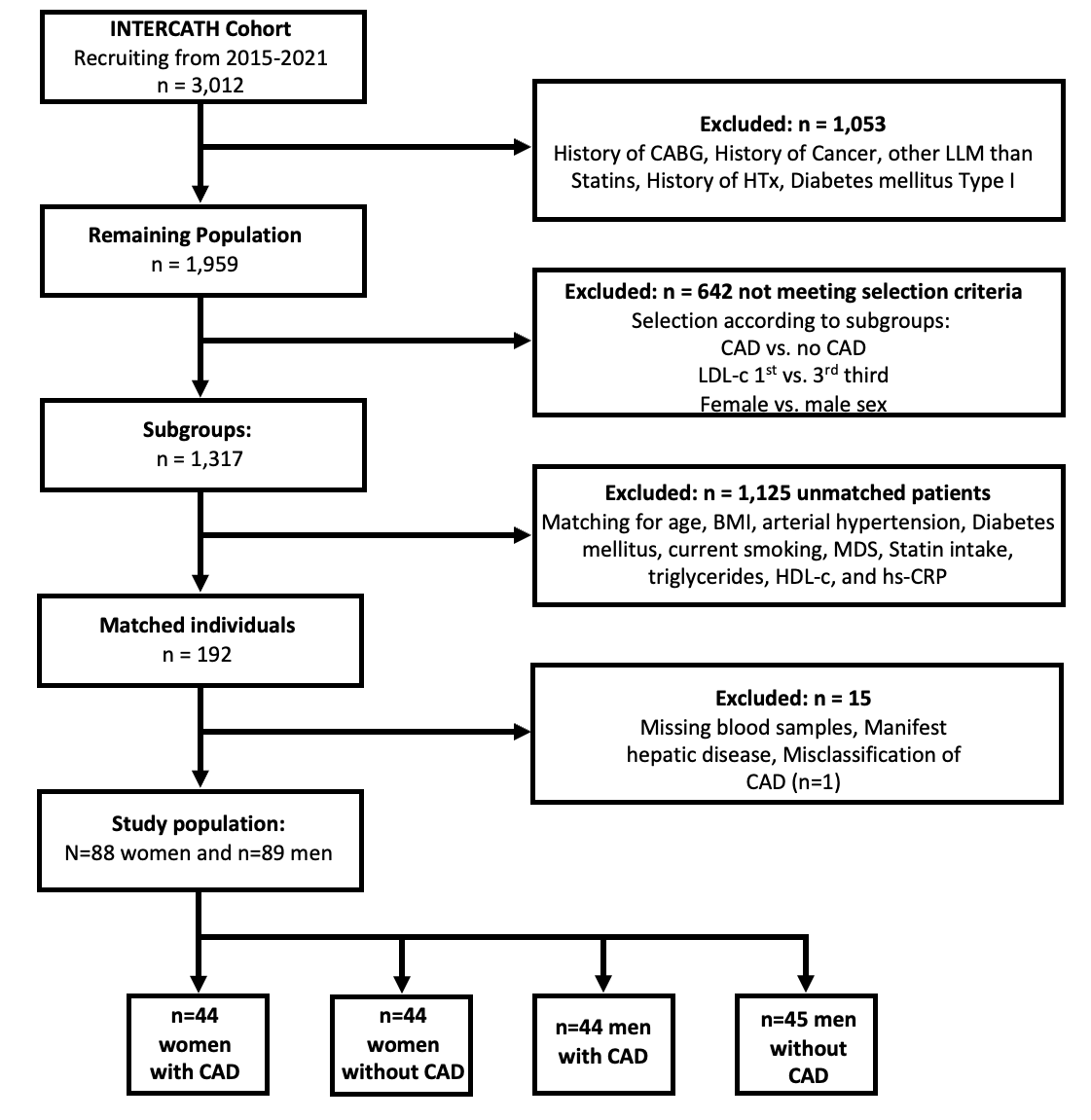
**

*CABG* Coronary artery bypass graft; *CAD* Coronary artery disease; *HDL-c* High-density lipoprotein cholesterol; *hs-CRP* high-sensitivity C-reactive protein; *HTx* Heart transplantation; *LDL-c* Low-density lipoprotein cholesterol; *LLM* Lipid lowering medication; *MDS* Mediterranean diet score.

***Table S1: Determined bile acid species.***

| **Full name** | **Abbreviation** | **HMDB** |
| --- | --- | --- |
| 7-Ketodeoxycholic acid | 7-oxo-CA | HMDB0000502 |
| 7-Ketochenodeoxycholic acid | 7-oxo-CDCA | HMDB00467 |
| Lithocholic acid | LCA | HMDB0000761 |
| Isodeoxycholic acid | iso DCA | HMDB0002536 |
| 3-Oxo-deoxycholic acid | 3-oxo-DCA | HMDB62742 |
| Glycochyodeoxycholic acid | GDHCA | HMDB0304944 |
| Glycohyodeoxycholic acid | GHCA | HMDB0242179 |
| Lithocholate 3-sulfate | LCA-3S | HMDB0242165 |
| glycolithocholic acid – 3- sulfate | GLCA-3S | HMDB0002639 |
| taurolithocholic acid - 3 sulfate | TLCA-3S | HMDB0002580 |
| Taurocholic acid | TCA | HMDB0000036 |
| Ursocholic aicd | UCA | HMDB0000917 |
| Glycochenodeoxycholic | GCDCA | HMDB0000637 |
| Glycodeoxycholic acid | GDCA | HMDB0000631 |
| taurochenodeoxycholic | TCDCA | HMDB0000951 |
| Taurodeoxycholic acid | TDCA | HMDB0000896 |
| Chenodeoxycholic | CDCA | HMDB0000518 |
| Deoxycholic acid | DCA | HMDB0000626 |
| Glycolithocholic acid | GLCA | HMDB0000698 |
| Glycolcholic acid | GCA | HMDB0000138 |
| Cholic acid | CA | HMDB0000619 |

*HMDB* Human Metabolome DataBase.

***Table S2: Baseline characteristics according to sex, presence or absence of CAD and LDL-c third.***

|  | **Female** | | | | **Male** | | | |  |
| --- | --- | --- | --- | --- | --- | --- | --- | --- | --- |
|  | **CAD & LDL-c**  **1^st^ third**  **(N=23)** | **no CAD & LDL-c**  **1^st^ third**  **(N=22)** | **CAD & LDL-c**  **3^rd^ third**  **(N=21)** | **no CAD & LDL-c**  **3^rd^ third**  **(N=22)** | **CAD & LDL-c**  **1^st^ third**  **(N=21)** | **no CAD & LDL-c**  **1^st^ third**  **(N=23)** | **CAD & LDL-c**  **3^rd^ third**  **(N=23)** | **no CAD & LDL-c**  **3^rd^ third**  **(N=22)** | **p-value** |
| **Demographics and comorbidities** |  |  |  |  |  |  |  |  |  |
| Age  (years) | 72.4 (63.6, 79.4) | 70.8 (59.4, 77.2) | 75.3 (64.9, 77.8) | 65.4 (58.5, 73.5) | 70.9 (67.4, 77.6) | 62.5 (57.8, 76.2) | 72.3 (62.5, 76.7) | 72.2 (62.7, 77.3) | 0.41 |
| BMI  (kg/m^2^) | 25.4 (23.4, 29.4) | 24.5 (21.6, 27.9) | 27.2 (23.9, 30.9) | 25.2 (22.8, 28.3) | 26.3 (24.1, 27.5) | 26.3 (24.9, 29.6) | 26.3 (24.2, 28.8) | 26.5 (24.3, 30.6) | 0.75 |
| Hypertension  (%) | 22 (95.7) | 21 (95.5) | 20 (95.2) | 21 (95.5) | 20 (95.2) | 21 (91.3) | 22 (95.7) | 19 (86.4) | 0.89 |
| Diabetes mellitus  (%) | 2 (8.7) | 1 (4.6) | 2 (9.5) | 1 (4.6) | 2 (9.5) | 2 (8.7) | 2 (8.7) | 1 (4.6) | 0.99 |
| Current Smoking  (%) | 12 (52.2) | 14 (63.6) | 10 (47.6) | 10 (45.5) | 10 (47.6) | 13 (56.5) | 15 (65.2) | 12 (54.6) | 0.84 |
| MDS  (points) | 12.7 (10.8, 15.0) | 13.0 (11.1, 14.2) | 13.3 (11.8, 14.3) | 13.0 (10.5, 13.9) | 13.7 (12.0, 14.7) | 13.0 (11.3, 14.5) | 13.3 (11.8, 14.3) | 13.2 (11.3, 14.7) | 0.94 |
| Intake of statins  (%) | 7 (30.4) | 4 (18.2) | 5 (23.8) | 6 (27.3) | 5 (23.8) | 3 (13.0) | 4 (17.4) | 4 (18.2) | 0.87 |
| **Laboratory values** |  |  |  |  |  |  |  |  |  |
| HDL-c  (mg/dL) | 53.0 (44.5, 60.0) | 55.0 (42.5, 67.8) | 55.0 (48.0, 66.0) | 54.0 (46.6, 59.8) | 54.0 (49.0, 59.0) | 45.0 (38.5, 64.5) | 52.0 (39.5, 58.5) | 51.0 (43.0, 62.8) | 0.53 |
| Triglycerides  (mg/dL) | 104.0 (77.5, 124.5) | 83.5 (65.0, 132.3) | 107.0 (78.0, 114.0) | 98.5 (81.5, 129.8) | 80.0 (70.0, 120.0) | 94.0 (57.0, 113.0) | 104.0 (75.5, 129.0) | 85.0 (71.0, 102.0) | 0.56 |
| hs-CRP  (mg/L) | 0.2 (0.1, 0.7) | 0.5 (0.2, 0.9) | 0.3 (0.2, 0.6) | 0.3 (0.1, 0.5) | 0.2 (0.1, 1.4) | 0.2 (0.1, 1.3) | 0.4 (0.1, 0.7) | 0.24 (0.2, 0.4) | 0.84 |

Categorical variables are shown as absolute numbers and percentages and were compared by fisher’s exact test. Continuous variables are described by median and the 25th percentile/75th percentile and were compared by the Mann Whitney test. *BMI* body-mass index; *CAD* coronary artery disease; *HDL-c* high-density lipoprotein cholesterol; *hs-CRP* high-sensitivity C-reactive protein; *MDS* Mediterranean diet score; *LDL-c* low-density lipoprotein cholesterol.
